# The Contribution of Membrane Vesicles to Bacterial Pathogenicity in Cystic Fibrosis Infections and Healthcare Associated Pneumonia

**DOI:** 10.3389/fmicb.2020.00630

**Published:** 2020-04-09

**Authors:** Jolien Vitse, Bart Devreese

**Affiliations:** Laboratory of Microbiology, Department of Biochemistry and Microbiology, Ghent University, Ghent, Belgium

**Keywords:** bacteria, lung infection, pneumonia, cystic fibrosis, pathogenesis, membrane vesicles, antimicrobial resistance

## Abstract

Almost all bacteria secrete spherical membranous nanoparticles, also referred to as membrane vesicles (MVs). A variety of MV types exist, ranging from 20 to 400 nm in diameter, each with their own formation routes. The most well-known vesicles are the outer membrane vesicles (OMVs) which are formed by budding from the outer membrane in Gram-negative bacteria. Recently, other types of MVs have been discovered and described, including outer-inner membrane vesicles (OIMVs) and cytoplasmic membrane vesicles (CMVs). The former are mainly formed by a process termed endolysin-triggered cell lysis in Gram-negative bacteria, the latter are formed by Gram-positive bacteria. MVs carry a wide range of cargo, such as nucleic acids, virulence factors and antibiotic resistance components. Moreover, they are involved in a multitude of biological processes that increase bacterial pathogenicity. In this review, we discuss the functional aspects of MVs secreted by bacteria associated with cystic fibrosis and nosocomial pneumonia. We mainly focus on how MVs are involved in virulence, antibiotic resistance, biofilm development and inflammation that consequently aid these bacterial infections.

## Diversity of Membrane Vesicles

Secretion of membrane vesicles (MVs) is a common feature in almost all bacteria. Over the last decade, studies of the MV content and physiological analyses revealed that bacterial MVs are involved in a wide range of biological processes, including virulence, antibiotic resistance, horizontal gene transfer, cell-cell communication, iron scavenging, nutrient acquisition, modulating the host immune system, and protection against phage infections (Manning and Kuehn, 2011; Elhenawy et al., 2014; Haurat et al., 2015; Schwechheimer and Kuehn, 2015). MVs protect their cargo against extracellular degrading enzymes, enable long distance transport, facilitate efficient delivery to target host cells and deliver their cargo at increased concentrations, which makes them perfectly suited for secretion or uptake of nucleic acids, proteins and other biomolecules (Bonnington and Kuehn, 2014).

Early MV research focused on one specific type of MVs, namely the outer membrane vesicles (OMVs). They are formed by pinching of the outer membrane (OM) of Gram-negative bacteria and are enriched for OM proteins and periplasmic components. Several mechanisms of OMV production have already been described (Mashburn-Warren et al., 2008, 2009; Deatherage et al., 2009; Tashiro et al., 2010; Schwechheimer et al., 2014; Haurat et al., 2015; Schwechheimer and Kuehn, 2015; Roier et al., 2016a, b; Florez et al., 2017; Horspool and Schertzer, 2018; Cooke et al., 2019). Up until recently, OMVs were the only known MVs secreted by Gram-negative bacteria. Today, several other types of MVs have been discovered and described, for example outer-inner membrane vesicles (OIMVs) and cytoplasmic membrane vesicles (CMVs). Compelling evidence was provided that OIMV production in *Pseudomonas aeruginosa* is dependent on the action of a prophage endolysin, resulting in a phenomenon referred to as phage endolysin-triggered cell lysis (Turnbull et al., 2016). Lastly, it was thought that MVs only arose from Gram-negative bacteria, but the observation of vesiculation in several Gram-positive bacteria changed that view (Brown et al., 2015). Since Gram-positive bacteria do not possess an OM, MVs released by these bacteria are termed cytoplasmic membrane vesicles (CMVs). CMV release can be triggered by phage endolysin-triggered cell lysis as well, as was demonstrated in *Bacillus subtilis* and *Staphylococcus aureus* (Toyofuku et al., 2017; Andreoni et al., 2019). The different MV types each with their own formation routes were recently reviewed by Toyofuku et al. (2019). It should be recognized that some forms of MVs were only recently described. Previous studies may have ignored them and their results could thus refer to a mixture of different types of MVs instead of pure OMVs. Consequently, in this review, we use the term MVs to cover all types.

## Membrane Vesicles from Bacteria Associated with Cystic Fibrosis and Nosocomial Pneumonia

**FIGURE 1:**
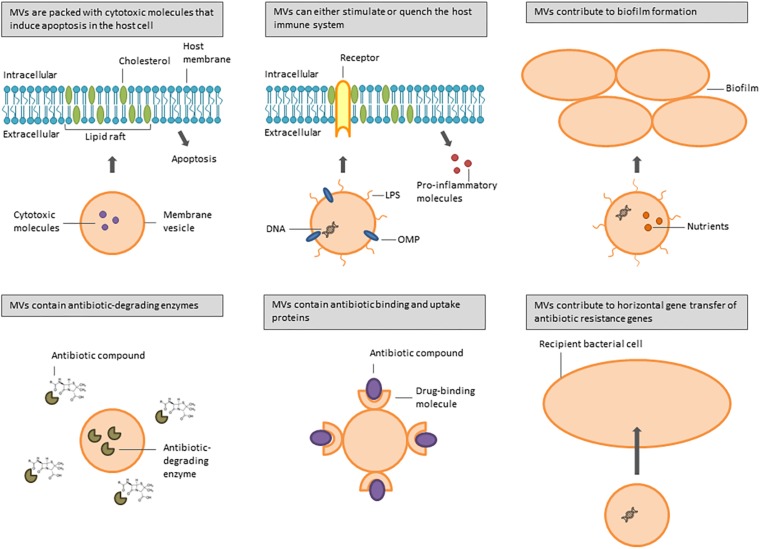
Overview of functions of MVs secreted by bacteria involved in CF and nosocomial lung infections.

Several bacterial species are associated with airway infections in immunocompromised and cystic fibrosis (CF) patients, including the Gram-negative bacteria *P. aeruginosa*, *Acinetobacter baumannii*, *Stenotrophomonas maltophilia*, *Klebsiella pneumoniae*, *Haemophilus influenzae*, *Moraxella catarrhalis*, and *Burkholderia* spp., and the Gram-positive bacteria *Streptococcus pneumoniae* and *S. aureus*. They all produce MVs involved in a multitude of biological processes that increase bacterial pathogenicity (Figure 1). Table 1 gives an overview of the different pathogenicity mechanisms associated with MVs secreted by these pathogens.

**TABLE 1:** Overview of the impact on membrane vesicles produced by species associated with CF and nosocomial pneumonia on pathogenicity, based on factors identified in MVs.

Impact on pathogenicity	Species involved	Molecules involved
Virulence factors contained in OMVs	*P. aeruginosa*, *A. baumannii*, *B. cepacia*, *S. pneumoniae*, *S. aureus*	Cif^Pa^, Phospholipase C^Pa, Ab^, alkaline phosphatase^Pa^, proteases^Pa, Ab, Bc^, OmpA^Ab^, haemolysins^Ab^, leukotoxins^Ab^, lipases^Bc^, pneumolysin^Sp^, α-toxin^Sa^, protein A^Sa^, exfoliative toxin A^Sa^
Increase of antibiotic resistance	*A. baumannii*, *S. maltophilia*, *H. influenzae*, *M. catarrhalis*	β-lactamase^Ab, Sm, Hi, Mc^
Adhesion to lung epithelial cells and macrophages	*P. aeruginosa*, *A. baumannii*, *H. influenzae*, *S. aureus*	PaAP^Pa^, LPS^Pa^, EF-Tu^Ab^
Biofilm formation	*P. aeruginosa*, *S. aureus*	eDNA^Pa^, LPS^Pa^, peptidoglycan^Pa^
Immunomodulatory effect (stimulation)	*P. aeruginosa*, *S. maltophilia*, *H. influenzae*, *M. catarrhalis*, *K. pneumoniae*, *S. pneumoniae*	FliC^Pa^
Immunomodulatory effect (quenching)	*P. aeruginosa*, *K. pneumoniae*, *S. pneumoniae*	sRNA^Pa^, porin-loss^Kp^, DNase^Sp^

*Indices indicate the species to which each molecule belongs, for references, see main text.*

### MV Cargo

The cargo of MVs can be very versatile, from nucleic acids to virulence factors and antibiotic resistance components. Depending on the different MV formation routes, different types of cargo are selected. Logically, OMVs tend to be enriched for OM proteins and periplasmic components, while OIMVs and CMVs are enriched for DNA, RNA and cytoplasmic proteins. Extracellular oriented membrane components such as lipopolysaccharides (LPS) might be expected in any of these vesicles.

*Pseudomonas aeruginosa* MVs released during the exponential growth phase were specifically packed with chromosomal DNA, consisting of specific chromosomal regions encoding proteins involved in stress response, virulence, metabolism and antibiotic resistance. These MVs can transfer DNA or sRNA into cultured lung epithelial or bronchial cells and by doing so, modulate the host cell responses (Koeppen et al., 2016; Bitto et al., 2017). Next to nucleic acids, *P. aeruginosa* MVs tend to pack several proteinaceous virulence factors, including hemolytic phospholipase C, alkaline phosphatase and the cystic fibrosis transmembrane conductance regulator (CFTR) inhibitory factor Cif. The latter inhibits CFTR-mediated chlorine secretion in the airways resulting in a decreased mucociliary clearance (Kadurugamuwa and Beveridge, 1995; Bomberger et al., 2009). Other proteins identified in *P. aeruginosa* MVs are involved in proteolysis, antibiotic resistance, and bacteria-host interactions (Choi et al., 2011).

*Acinetobacter baumannii* MVs are likewise enriched for several virulence factors, such as proteases, phospholipase C, hemolysins and leukotoxins. The MVs seem to interact with and deliver their content to host cells. In addition, the cytotoxic outer membrane protein A (OmpA) was identified in these MVs as well. OmpA, an important virulence factor in *A. baumannii*, is targeted to the mitochondria in epithelial cells and consequently induces apoptosis in these cells (Kwon et al., 2009; Jha et al., 2017). Moreover, Dallo et al. (2012) discovered that the elongation factor Tu (EF-Tu) is associated with *A. baumannii* MVs and interacts with macrophages through its binding with fibronectin.

*Burkholderia cepacia* MVs contain peptidoglycan-degrading enzymes together with a variety of virulence factors, including lipases, phospholipases and proteases (Allan et al., 2003). In *S. pneumoniae*, MVs are mainly enriched for lipoproteins as well as the cytosolic pore-forming toxin pneumolysin (Olaya-Abril et al., 2014). Further, MVs from clinical *S. aureus* isolates have a different cytotoxic activity on host cells, depending on their MV proteomes. The exfoliative toxin A (ETA) was specifically enriched in MVs with a high cytotoxic activity (Jeon et al., 2016).

### Interaction of MVs With Lung Epithelial Cells and Macrophages

The presence of MVs in the lungs of patients with severe lung infections (Bomberger et al., 2009) and the fact that MVs can carry virulence factors, may point to a role of these MVs in the infection process. A few examples of MV-host cell interactions were already mentioned above. Additionally, Bauman and Kuehn (2009) discovered that MVs from a *P. aeruginosa* CF isolate interact with human lung cells and were internalized in a time- and dose-dependent manner. These bacteria secrete PaAP, an aminopeptidase mainly present on the surface of MVs and was found to be important for the association of the MVs with the lung cells. Further, MVs secreted by *P. aeruginosa* interact with cholesterol-rich lipid rafts in the apical membrane of lung epithelial cells. By doing so, *P. aeruginosa* is able to deliver the virulence factor Cif to the cytoplasm of the host cell and consequently reduce the CFTR chloride secretion (Bomberger et al., 2009, 2011; Ballok et al., 2014; O’Donoghue and Krachler, 2016). Lowering the cholesterol content of CF airway epithelial cells (Phe508del) by cyclodextrin lowers the impact of *P. aeruginosa* vesicles on Cl^–^ secretion after lumacaftor treatment (Barnaby et al., 2019).

Likewise, purified vesicles secreted by Non-Typeable *H. influenzae* (NTHi) co-localize with caveolin, a protein involved in endocytosis. This indicates that the uptake of MVs is mediated by caveolae, which are cholesterol-rich lipid rafts. On top of that, the interactions of these MVs with epithelial cells resulted in the release of the immunomodulatory cytokine interleukin-8 (IL-8) and the antimicrobial peptide LL-37 (Sharpe et al., 2011). Further, NTHi released MVs while infecting primary respiratory epithelial cells grown at the air-liquid interface. Transmission electron microscopy (TEM) revealed that these MVs directly interact with the host-cell membranes. However, the role of these vesicles during NTHi infection is yet to be determined (Ren et al., 2012).

Membrane vesicles released by *S. aureus* also fuse in a cholesterol-dependent manner with the plasma membrane of human cells, resulting in the delivery of α-toxin. This toxin, also known as α-hemolysin (HIa), is a 33 kDa pore-forming protein and a key virulence factor capable of lysing human cells and the induction of apoptosis in T-lymphocytes. Furthermore, this MV-associated protein is involved in HeLa cell cytotoxicity and erythrocyte lysis (Thay et al., 2013). Gurung et al. (2011) discovered that *S. aureus* MVs interact with the plasma membrane of human cells through a cholesterol-rich micro-domain as well. The MVs subsequently delivered the immunoglobulin G-binding protein (protein A) and induced apoptosis of HEp-2 cells in a dose-dependent manner.

The same mechanism is true for *A. baumannii*. MVs from *A. baumannii* ATCC 19606(T) interacted with lipid rafts in the plasma membrane of human cells and induced apoptosis in the host cells. The effect was lost when MVs secreted by the Δ*ompA* mutant strain were studied. Suggesting a role of the MV-associated virulence factor OmpA in host cell-death (Jin et al., 2011).

While most of these studies were performed in *in vitro* systems, a few studies in mice models also indicate the impact of bacterial membrane vesicles in lungs. Jang et al. (2015) injected *Escherichia coli* derived vesicles intraperitoneally and showed that these vesicles spread into lungs. Proteins from *A. baumannii* MVs delivered intranasally in mice were detected in the lungs and provoked an immune response (Marion et al., 2019).

### Effect of Antibiotics on MV Secretion and Function

Devos et al. (2015) demonstrated that antibiotic stress leads to an increased secretion of MVs in *S. maltophilia* 44/98, suggesting that this could have potential implications on the function of these MVs. Indeed, the exposure of *S. maltophilia* to the β-lactam antibiotic imipenem led to an increased secretion of MVs comprising two chromosomally encoded β-lactamases. These β-lactamase-containing MVs are capable of mediating extracellular β-lactam degradation and consequently enhance the β-lactam tolerance of other CF pathogens, including *P. aeruginosa* and *Burkholderia cenocepacia* (Devos et al., 2016). Several other bacteria exposed to β-lactam antibiotics release MVs containing functional β-lactamases as well. MVs secreted by β-lactam-resistant *M. catarrhalis* and NTHi can hydrolyze amoxicillin and consequently protect co-localized species, such as Group A *Streptococci*, *S. pneumoniae* and *H. influenzae*, from killing by amoxicillin (Schaar et al., 2014). In this regard, imipenem-treatment of *A. baumannii* resulted in an elevated secretion of MVs, containing β-lactamase OXA-23, with a higher cytotoxicity toward A549 human lung cells (Yun et al., 2018).

Moreover, Allan and Beveridge (2003) discovered that the treatment of *P. aeruginosa* PAO1 with the aminoglycoside antibiotic gentamicin resulted in the release of MVs containing gentamicin and peptidoglycan hydrolase. By becoming bactericidal, these MVs are capable of killing group IIIa *B. cepacia.* Also *B. subtilis* 168 and *S. aureus* D_2_C were affected by these type of MVs and *Listeria monocytogenes* ATCC 19113 was susceptible to a lesser extent (MacDonald and Beveridge, 2002).

On another note, *P. aeruginosa* infections treated with tobramycin led to a reduced secretion of MV-associated virulence factors, including AprA, which is an alkaline protease that reduces CFTR-mediated chloride secretion. AprA is essential for the survival of *P. aeruginosa* in the lungs as it inhibits the bacterial clearance (Koeppen et al., 2019).

### Interspecies Interactions of MVs

Cell-cell communication between bacteria during lung infections is key to the survival of the infecting species, e.g., in the mixed species biofilm in the lungs of patients with CF. Most interspecies interactions have been studied *in vitro*, but some advanced three-dimensional lung cell culture models approve some of the findings performed in co-culturing experiments (Rodriguez-Sevilla et al., 2018). Communication via MVs can mediate changes in the expression of biofilm-related genes, or protect other species from antibiotics and host defense mechanisms. Kadurugamuwa and Beveridge (1999) discovered that MVs secreted by *P. aeruginosa* and *Shigella flexneri* can be integrated into the membrane of other Gram-negative bacteria. Moreover, the MVs of two carbapenem-resistant clinical strains of *A. baumannii* harboring the plasmid-borne *bla*_*OXA–24*_ gene, encoding a β-lactamase, were capable of protecting a carbapenem-susceptible *A. baumannii* strain. The presence of these plasmids in the carbapenem-susceptible strain suggests that *A. baumannii* releases MVs to mediate horizontal gene transfer of antibiotic-resistance genes (Rumbo et al., 2011). In addition, vesicles released by *A. baumannii* can mediate gene transfer of the *bla*_*NDM–1*_ and *aac*(6′)*-Ib-cr* genes to other *A. baumannii* and *E. coli* recipient cells (Chatterjee et al., 2017). Next to this, the MVs of *M. catarrhalis* mediated protection of *H. influenzae* against the complement system during infection (Tan et al., 2007).

### MVs in Biofilm

Single- or poly-microbial infections in the lungs are mostly paired with biofilm formation. Biofilms are characterized by a thick layer of bacterial cells formed by the co-operation of several virulence factors, including flagella, fimbriae, pili and LPS, and is surrounded by a self-producing extracellular matrix consisting of polysaccharides, proteins, lipids and nucleic acids (O’Toole et al., 2000; Flemming and Wingender, 2010).

Membrane vesicles are very abundant in biofilm related infections (Schooling and Beveridge, 2006; Toyofuku et al., 2012; Grande et al., 2015). Indeed, the proteome analysis of *P. aeruginosa* biofilms revealed that MV-associated proteins contribute to more or less 20% of the whole matrix proteome. The MV-related proteins identified were OM enzymes and proteins involved in the transport of small molecules, the uptake of iron and antibiotic resistance (Couto et al., 2015). In addition, vesicles purified from late-stage *P. aeruginosa* biofilms are enriched for drug-binding proteins, which makes the bacterial species inside these biofilms even better protected against antibiotics (Park et al., 2014). It has been suggested further that MVs secreted by *P. aeruginosa* are under the control of the quorum sensing system and supply the forming biofilm with extracellular DNA (eDNA) and LPS (Nakamura et al., 2008). Moreover, studies in *P. aeruginosa* biofilms revealed that MVs secreted by one species are able to lyse neighboring bacteria in order to release nutrients as a source for growth and eDNA to build the biofilm (Beveridge et al., 1997). However, following research revealed that the MVs themselves are actually incorporated in the biofilm matrix (Schooling and Beveridge, 2006). This was seen in *Francisella* biofilms as well (van Hoek, 2013).

He et al. (2017, 2019) discovered that MV secretion in methicillin-resistant *S. aureus* (MRSA) was correlated with biofilm formation during improper vancomycin chemotherapy. They also demonstrated that the treatment of MRSA with β-lactam antibiotics induces biofilm formation as a consequence of MV secretion with a higher hydrophobicity.

### Immunomodulatory Effects of MVs

Several reports indicated that MVs can exert immunomodulatory effects and aid in pathogenesis, a few examples were already mentioned. MVs secreted by *P. aeruginosa* are able to activate an IL-8 response by lung epithelial cells, as was seen in NTHi as well (see above). In this way, vesicles could contribute to inflammation (Bauman and Kuehn, 2006). It was demonstrated that *P. aeruginosa* MVs induce the upregulation of pro-inflammatory cytokines in macrophages. The response was even greater compared to the induction of cytokines with purified LPS. This study revealed that MV-associated LPS is required for binding to the macrophages and the internalization is mediated by the protein content of the MVs. Interestingly, they also showed that intensity of IL-8 response is strain dependent and was the highest in a CF isolate (compared to the acute PAO1 strain). In addition, flagellin (FliC) was identified as one of the most abundant proteins in *P. aeruginosa* MVs and is responsible for the cytokine release in macrophages (Ellis et al., 2010). Moreover, MVs from *P. aeruginosa* cause pulmonary inflammation in a bacteria-free *in vivo* setting. The MVs caused a time- and dose-dependent pulmonary inflammation comparable to the response of live bacteria (Park et al., 2013). *A. baumannii* MVs provoked an inflammatory response *in vivo* (mouse model) resulting in secretion of cytokines and chemokines, mediated via Toll-like receptors (Marion et al., 2019). Although LPS embedded in MVs might be very important in this inflammatory response, it should be noted that MVs obtained from LPS-free *Neisseria meningitidis* did not provoke a significant different response than LPS positive MVs, indicating that other components like OMPs can function as complement activators (Bjerre et al., 2002).

Similarly, *S. pneumoniae* MVs are internalized into A549 lung epithelial cells and human monocyte-derived dendritic cells and result in pro-inflammatory cytokine responses (Codemo et al., 2018). Also *M. catarrhalis* MVs are internalized by human epithelial cells and induce an inflammatory response. On the other hand, proteomic analyses revealed that these MVs contain factors that aid these bacteria to evade the host defense system as well (Schaar et al., 2011). Further, MVs secreted by respiratory pathogens, including NTHi, *M. catarrhalis*, and *P. aeruginosa*, induce a strong pro-inflammatory response by naïve THP-1 macrophages (Volgers et al., 2017b). Regarding to this, N-acetyl-L-cysteine (NAC), a mucolytic that reduces the production of thick mucus, induced the release of pro-inflammatory MVs by these respiratory pathogens, but decreased the release of pro-inflammatory cytokines in macrophages (Volgers et al., 2017a). Moreover, MVs secreted by *S. maltophilia* ATCC 13637 were cytotoxic to A549 epithelial cells and induced the expression of pro-inflammatory cytokine and chemokine genes in these lung cells (Kim et al., 2016). MVs originated from *K. pneumoniae* ATCC 13883 likewise induced changes in the expression of immune-related genes in epithelial cells. The expression of genes encoding for IL-8, IL-1b, MIP-1α, HMOX1, HSPA1A, and IL-24, was increased after treatment of these cells with *K. pneumoniae* MVs (Lee et al., 2012; You et al., 2019). Further, several Gram-negative bacteria bind to epithelial cells through lipid rafts and deliver peptidoglycan-containing MVs to the intracellular sensor NOD1 to promote inflammation (Kaparakis et al., 2010).

Koeppen et al. (2016) discovered a novel mechanism of host-pathogen interaction mediated by MVs secreted by *P. aeruginosa*. The MVs are packed with sRNA molecules that bind to mRNA inside human lung cells and in this way quench the human immune response. Similarly, an extracellular DNase was identified in MVs from *S. pneumoniae* that blocks neutrophil activity and helps to evade the host innate immune response (Jhelum et al., 2018). Porin-loss, which is common in antibiotic-resistant strains of *K. pneumoniae*, impacts the MV composition and the host-inflammatory response. MVs lacking several OM porins were less likely to elicit the secretion of pro-inflammatory cytokines in macrophages. Antibiotic resistance resulting in porin-loss in *K. pneumoniae* can thus have an impact on the survival of this pathogen (Turner et al., 2015). Further, MVs from Gram-negative bacteria induced vitronectin in mouse lungs and in A549 epithelial cells, which is released into the bronchoalveolar space and mediates protection against complement-mediated clearance (Paulsson et al., 2018).

The immunomodulatory effects of MVs can be useful to protect patients from bacterial infections. A vaccine based on detergent-extracted OMVs originating from the pathogenic bacterium *Neisseria meningitides*, complemented with recombinant proteins, has recently been approved and used to protect people against meningitis B. Several traits, such as the overexpression of certain antigens or the modification of the LPS reactogenicity, can be altered by genetically engineering the OMV-producing bacteria to yield a vaccine that meets the specific needs (van der Pol et al., 2015). Furthermore, active immunization of mice with *P. aeruginosa* MVs resulted in mice that were protected from *P. aeruginosa* infections (Zhang et al., 2018). Also NTHi, *K. pneumoniae* and *S. aureus* MVs are potential vaccine candidates, as was demonstrated in several studies (Lee et al., 2015; Roier et al., 2015; Winter and Barenkamp, 2017; Askarian et al., 2018).

## Concluding Remarks

Bacteria associated with lung infections are increasingly posing a threat for the public health worldwide. In particular, the impact on CF and immunocompromised patients is concerning. Therefore, it is crucial to shed more light on the mechanisms these bacteria use to increase their pathogenicity. MVs were discussed to play an important role herein. By targeting MV-associated components that are involved in the interaction of these vesicles with human lung cells or macrophages, new therapeutic options to treat these infections could arise. Furthermore, the immunomodulatory effects of MVs could be exploited to produce vaccines leading to the protection of patients against the infecting bacteria. Taken together, it is important to further investigate the role of MVs during bacterial infection and the use of MVs to eventually combat these infections. Importantly, more *in vivo* studies are required to investigate the real impact of MVs on the progression of disease.

## Author Contributions

JV wrote the manuscript. BD was the supervisor of JV and edited and corrected the manuscript together with JV.

## Conflict of Interest

The authors declare that the research was conducted in the absence of any commercial or financial relationships that could be construed as a potential conflict of interest.
